# Künstliche Intelligenz in chirurgischen Disziplinen: Einsatz, Nutzen und Potenzial – ein Delphi-Expertenkonsensus

**DOI:** 10.1007/s00120-026-02778-8

**Published:** 2026-01-30

**Authors:** G. Duwe, K. Moench, V. Kauth, M. Angeloni, J. Eckhoff, M. Görtz, S. Hoefert, T. D. Kocar, L. Kollitsch, S. Mehralivand, D. Mercier, J. Rudolph, J. Rueckel, R. Schönhof, M. Sondermann, CAJ von Klot, A. Zamzow, J. P. Struck, H. Borgmann

**Affiliations:** 1 Klinik und Poliklinik für Urologie und Kinderurologie, Universitätsmedizin der Johannes Gutenberg-Universität Mainz, Langenbeckstr. 1, 55131 Mainz, Deutschland; 2 Pathologisches Institut, Universitätsklinikum der Friedrich-Alexander-Universität Erlangen, Erlangen, Deutschland; 3 Klinik für Allgemein‑, Viszeral‑, Thorax- und Transplantationschirurgie, Universitätsklinikum Köln, Köln, Deutschland; 4 Nachwuchs-Klinische Kooperationseinheit „Multiparametrische Methoden zur Früherkennung von Prostatakrebs“, Deutsches Krebsforschungszentrum (DKFZ), Heidelberg, Deutschland; 5 Klinik und Poliklinik für Mund‑, Kiefer- und Gesichtschirurgie, Universitätsklinikum Tübingen, Tübingen, Deutschland; 6 Institut für Geriatrische Forschung, Universitätsklinikum Ulm, Ulm, Deutschland; 7 Abteilung für Urologie und Andrologie, Klinik Donaustadt, Wien, Österreich; 8 Klinik und Poliklinik für Urologie, Universitätsklinikum Carl Gustav Carus Dresden, Dresden, Deutschland; 9 Deutsches Forschungszentrum für Künstliche Intelligenz (DFKI), Kaiserslautern, Deutschland; 10 Klinik und Poliklinik für Radiologie, LMU Klinikum, LMU München, München, Deutschland; 11 Institut für Diagnostische und Interventionelle Neuroradiologie, LMU Klinikum, LMU München, München, Deutschland; 12 Klinik für Urologie und Urologische Onkologie, Medizinische Hochschule Hannover, Hannover, Deutschland; 13 Institut für Medizinische Lehre und Ausbildungsforschung, Julius-Maximilians-Universität Würzburg, Würzburg, Deutschland; 14 Klinik für Urologie und Kinderurologie, Universitätsklinikum Brandenburg an der Havel, Brandenburg an der Havel, Deutschland; 15 Surgical Artificial Intelligence & Innovation Laboratory (SAIIL), Massachusetts General Hospital, Boston, USA; 16 Urologische Klinik, Universitätsklinikum Heidelberg, Heidelberg, Deutschland; 17 Fakultät für Gesundheitswissenschaften Brandenburg, Medizinische Hochschule Brandenburg Theodor Fontane, Brandenburg, Deutschland

**Keywords:** Künstliche Intelligenz, Chirurgie, Delphi-Konsensuskonferenz, Rechtliche Rahmenbedingungen, Digitalisierung, Artificial intelligence, Surgery, Delphi consensus conference, Legal framework, Digitalization

## Abstract

**Hintergrund:**

Künstliche Intelligenz (KI) in der chirurgischen Medizin hat das Potenzial, alle Behandlungsphasen im Sinne einer verbesserten Behandlungsqualität und Patientensicherheit zu unterstützen. Eine Expertengruppe hat im Rahmen einer Konsenskonferenz beim 2. Digital Health Summit in Brandenburg im August 2024 den aktuellen Stand und die erforderlichen Schritte zur erfolgreichen Integration von KI in chirurgische Fächer diskutiert.

**Methodik:**

Es wurde ein modifiziertes Delphi-Verfahren mit 16 interdisziplinären Forschern zum Themenkomplex KI in chirurgischen Disziplinen durchgeführt. In zwei Online-Meetings mit anschließenden Delphi-Umfragerunden (LimeSurvey) und einem abschließenden Hybrid-Meeting wurden von allen 16 Teilnehmern individuelle Stellungsnahmen erstellt, gemeinsam diskutiert und in Anlehnung an das AWMF-Regelwerk Leitlinien konsentiert.

**Ergebnisse:**

Aus 103 eingereichten Stellungsnahmen wurden 36 Statements zu Realität (*n* = 12), Utopie (*n* = 13) und Möglichkeiten der digitalen Transformation (*n* = 11) nach Diskussion und Modifikation konsentiert. Wir erzielten einen Konsens von mindestens 75 % in allen hier aufgeführten Stellungsnahmen, 6 der Stellungsnahmen erreichten mit 100 % Zustimmung starken Konsens.

**Schlussfolgerung:**

Die konsentierten Stellungsnahmen zeigen das große Potenzial von KI für die Verbesserung in allen Bereichen der Patientenversorgung in der chirurgischen Medizin. Herausforderungen wie fehlende Digitalisierungsstrukturen und rechtliche Rahmenbedingungen wurden identifiziert und praxisnahe Vorschläge zur Umsetzung entwickelt. Die Notwendigkeit einer interdisziplinären Zusammenarbeit zwischen Medizin, Politik und Wirtschaft wird betont, um das deutsche Gesundheitswesen national wie international wettbewerbsfähig und zukunftssicher zu gestalten.

## Abstract (en)

**Background:**

Artificial intelligence (AI) in surgical disciplines has the potential to support all areas of patient care, with the goal of improving treatment quality and patient safety. A group of multidisciplinary experts discussed the current situation as well as steps required to successfully integrate AI into surgical disciplines in the context of a consensus conference at the second Digital Health Summit (Brandenburg an der Havel, Germany) in August 2024.

**Methods:**

A modified Delphi procedure was performed with 16 multidisciplinary physicians and scientists on the topic of AI in surgical disciplines and beyond. In two online meetings with subsequent Delphi survey rounds (LimeSurvey) and a final hybrid meeting, individual statements were contributed, discussed, and consented by all 16 participants based on current national clinical guidelines.

**Results:**

From a total of 103 submitted statements, 36 statements on reality (*n* = 12), utopia (*n* = 13), and opportunities for digital transformation (*n* = 11) were consented after discussion and modification. We achieved a consensus of at least 75% for all the statements presented, with six of the statements achieving a strong consensus of 100% agreement.

**Conclusion:**

The consensus statements show the great potential of AI for improving patient care in surgical disciplines. Challenges such as the lack of digitalization structures and legal frameworks were identified, and practice-oriented proposals for implementation were developed. The need for multidisciplinary cooperation between medical professionals, politics, and industry was emphasized in order to facilitate the German healthcare system remaining competitive for the future, both nationally and internationally.

Künstliche Intelligenz (KI) ist aus allen Bereichen des chirurgischen Alltags nicht mehr wegzudenken. Um unsere Patienten bestmöglich zu versorgen und als deutsches Gesundheitswesen international wettbewerbsfähig zu bleiben, gilt es, die Möglichkeiten und Herausforderungen zu benennen und in interdisziplinären Lösungen umzusetzen. 16 Experten beschreiben die aktuelle Situation, Zukunftswünsche sowie Ideen für den Weg in die digitale Transformation.

## Hintergrund

Die technischen Entwicklungen im Bereich der KI zeigen das Potenzial für einen Paradigmenwechsel in der chirurgischen Medizin auf allen Ebenen der ärztlichen Tätigkeiten [1]. Im Zuge einer chirurgischen Behandlung könnten KI-Anwendungen theoretisch alle Behandlungsschritte unterstützen: beginnend mit der radiologischen und pathologischen Diagnostik, Patientenaufklärung und edukativer Begleitung während der Behandlung, über eine intraoperative Unterstützung, z. B. durch autonome roboterassistierte Arbeitsschritte bis hin zur postoperativen Überwachung und Vorhersage von Behandlungsverläufen. Ziel aller Anwendungsbereiche ist es, die Behandlungsqualität und damit die Patientensicherheit zu verbessern.

Aufgrund der rasanten Entwicklung im Bereich der KI ist eine frühzeitige, strukturierte Zusammenarbeit zwischen den medizinischen, ärztlichen Anwendern, der Politik für die Verantwortung der rechtlichen Rahmenbedingungen und gesellschaftlichen Zielsetzungen sowie der freien Wirtschaft als Innovations- und Transformationstreiber notwendig. Hierfür haben wir als interdisziplinäre Expertengruppe für KI-Anwendungen in der Chirurgie im Rahmen des 2. Digital Health Summit am 30.08.2024 in Brandenburg an der Havel eine Konsenskonferenz nach Delphi-Maßgaben umgesetzt, um einen Status quo zur Realität sowie die notwendigen wissenschaftlichen und technischen Transformationsschritte, die eine skizzierte Utopie ermöglichen könnten, zu definieren [2]. Hierzu wurden neben den medizinischen Fragestellungen und Anforderungen insbesondere auch Aspekte zu der notwendigen „KI-Infrastruktur“ im Bereich Datenschutz, IT-Sicherheit und klinischer IT-Infrastruktur im Sinne der Digitalisierung diskutiert.

Mit den Ergebnissen dieser Konsensuskonferenz möchten wir allen beteiligten Akteuren in chirurgischen Disziplinen (und verwandten medizinischen Bereichen), Politik und Wirtschaft einen interdisziplinären, expertenvalidierten Überblick über den aktuellen Stand sowie die notwendigen Transformationsprozesse geben, um KI zukünftig in der chirurgischen Behandlung auf allen Ebenen erfolgreich und sinnvoll zu etablieren. Hierbei werden auch insbesondere berufsrechtliche und ethische Limitationen aufgezeigt, um aus ärztlicher Perspektive zielführende wissenschaftliche, technische und finanzielle Investitionen vorzuschlagen.

## Methodik

Wir führten ein modifiziertes Delphi-Verfahren mit 16 interdisziplinären Forschern zum Themenkomplex ,Künstliche Intelligenz in chirurgischen Disziplinen‘ durch. Die Zusammenstellung des Expertenpanels erfolgte durch E‑Mail-Einladungen an Erst‑/Letztautoren aus Deutschland und Österreich von Forschungsarbeiten auf PubMed zum Themenfeld. Die interdisziplinäre Zusammensetzung des Delphi-Panels erfolgte nach E‑Mail-Einladungen an Autorinnen und Autoren relevanter Forschungspublikationen der vergangenen 5 Jahre zum Themenfeld KI in der Medizin, die zuvor durch die Leitung des 2. Digital Health Summit identifiziert worden waren. Das Panel umfasste Experten aus klinisch-therapeutischen, diagnostischen und forschenden Bereichen – einschließlich Lehr- und Ausbildungsforschung – verschiedener deutscher Universitätskliniken, eines österreichischen Schwerpunktkrankenhauses sowie des Deutschen Forschungszentrums für Künstliche Intelligenz. Mit Ausnahme der Letztautoren sind sämtliche Mitglieder des Delphi-Panels zugleich Autorinnen und Autoren dieser Publikation. Die wissenschaftliche Methodik wurde in Zusammenarbeit mit dem Zentrum für Wissenstransfer „UroEvidence“ der Deutschen Gesellschaft für Urologie e. V. konzipiert. Die Delphi-Methode wurde in den 1950er-Jahren entwickelt und ist ein strukturiertes, mehrstufiges Befragungsverfahren zur Konsensbildung. Sie basiert auf der moderierten Sammlung anonymisierter Einschätzungen eines Expertenpanels [3].

Die Methodik des modifizierten Delphi-Verfahrens wurde im Voraus durch ein schriftliches Protokoll festgelegt. Alle eingeladenen Experten gaben ihre Interessenkonflikte offen an. Den Experten wurde ein Projektexposé mit allen relevanten Hintergrundinformationen bereitgestellt. Das Protokoll umfasste außerdem eine umfassende narrative Literaturrecherche zum Einsatz, Nutzen und Potenzial von KI in chirurgischen Disziplinen.

Es fanden insgesamt 2 Online-Meetings sowie 1 abschließendes Hybrid-Meeting (Präsenz + Online) mit den eingeladenen Experten statt. Im ersten Online-Meeting (via Microsoft Teams) erfolgte die Projektvorstellung sowie die Sammlung von Kernfragen und Argumenten. Nachfolgend entwickelten die Konsensusgruppenteilnehmer nach Literaturrecherche passende Konsensusstatements. Diese wurden durch die Konsensusgruppenleitung gebündelt und in die Bereiche Realität, Utopie und Transformation kategorisiert sowie thematisch gruppiert. Nachfolgend wurde die Konsensusumfrage auf der Plattform LimeSurvey an die Experten zur Abstimmung versandt. Der Grad der Zustimmung/Ablehnung zum Konsensusstatement erfolgte auf einer 5‑Punkte-Likert-Skala („stimme voll und ganz zu; stimme eher zu, stimme weder zu noch nicht zu, stimme eher nicht zu, stimme gar nicht zu“). Im zweiten Online-Meeting erfolgte die Vorstellung und Diskussion der bisherigen Konsensusergebnisse. Die Konsensusstatements wurden hier abhängig vom Zustimmungsgrad und der Diskussion belassen, geschärft, verändert oder verworfen. Nachfolgend wurde eine zweite Konsensusumfrage auf der Plattform LimeSurvey an die Experten zur Abstimmung versandt. Beim abschließenden Hybrid-Meeting wurden die Konsensusstatements dann final diskutiert, ggfs. adjustiert und final abgestimmt.

Der Grad der Konsensstärke in Abhängigkeit der Zustimmung der Experten zu den Konsensusstatements wurde in Anlehnung an das AWMF-Regelwerk Leitlinien wie folgt definiert:

- *starker Konsens*: Zustimmung von > 95 % der Teilnehmenden,
- *Konsens*: Zustimmung von > 75–95 % der Teilnehmenden,
- *mehrheitliche Zustimmung*: Zustimmung von > 50–75 % der Teilnehmenden,
- *keine mehrheitliche Zustimmung*: Zustimmung von ≤ 50 % der Teilnehmenden.

„Starker Konsens“ (> 95 % der Teilnehmenden) wurde aufgrund der Zusammensetzung der Gruppe aus 16 Experten nur dann erreicht, wenn alle 16 (100 %) eine Zustimmung angegeben haben. 15 Zustimmungen entsprechen hier 94 % und damit formal einem „Konsens“.

## Ergebnisse

Die erarbeiteten und konsentierten Statements beschäftigen sich zunächst mit der Realität (Status quo) der aktuellen KI-Nutzung in chirurgischen und angeschlossenen Disziplinen, definieren dann eine Utopie (maximale Potenzialausschöpfung) und beschreiben schließlich die aktuellen Möglichkeiten zur digitalen Transformation (vom Status quo zur maximalen Potenzialausschöpfung). Viele relevante Bereiche der chirurgischen Tätigkeiten werden thematisiert, jedoch besteht kein Anspruch auf Vollständigkeit.

### Realität (Status quo)

*Trotz* *rasanter wissenschaftlicher Weiterentwicklung von KI-Modellen und Erweiterung der Anwendungsgebiete mit Fokus auf chirurgische Sicherheit fehlen aktuell in Deutschland und europaweit essentielle strukturelle Voraussetzungen für die klinische Translation (technische, organisatorische, wirtschaftliche und regulatorische Voraussetzungen, sowie Akzeptanz- und Ausbildungsbarrieren). (KONSENS)*

Der begrenzte Zugang zu multizentrischen, umfassenden Datensätzen und die fehlende Interoperabilität zwischen chirurgischen Systemen erschweren den Datenaustausch und die Integration von KI-basierten Anwendungen, z. B. im Operationssaal. KI-Modelle müssen daher häufig an Hand von monozentrischen, spezifischen und weniger repräsentativen Datensätzen trainiert werden, was ihre Fähigkeit einschränkt, die klinische und demografische Vielfalt abzubilden [4, 5]. Die fehlende regulatorische Klarheit und unzureichende technische Infrastruktur zur nahtlosen Datenerfassung und -verarbeitung verhindern den Einsatz von KI, insbesondere bei zeitkritischen Entscheidungen im intraoperativen Setting.

*Für die nationale klinische Anwendung von KI-basierten Assistenzsystemen stellen die rechtlichen Verwaltungsstrukturen des Föderalismus für die flächendeckende Umsetzung von Vorgaben des Datenschutzes (mit einzelnen Datenschutzbeauftragten), der Informationssicherheit sowie der noch sehr heterogenen Digitalisierungsstandards eine aktuell komplexe politische, strukturelle Herausforderung dar. (STARKER KONSENS)*

Der aktuelle Einsatz von KI-Systemen im deutschen Gesundheitswesen erfolgt aktuell v. a. anhand einzelner Projekte in Kliniken bzw. Klinikverbunden. Somit muss jedes Krankenhaus eigenverantwortlich mit dem IT-Abteilungen sowie Datenschutzbeauftragten gesetzeskonforme und umsetzbare digitale Prozesse erarbeiten, die anschließend KI-Applikationen anwenden können. Für einen zukünftigen, effizienten, klinik- und länderübergreifenden Einsatz und Austausch von Daten für erfolgreiche KI-Systeme sollten in der Politik bundeseinheitliche Maßnahmen und Vorgaben erarbeitet werden, um diese bestehenden gesetzlichen Limitation zu verbessern [6–8].

*Die klinische Einführung von KI-Tools im medizinischen Bereich wird v.* *a. durch folgende Faktoren beeinflusst und aktuell verzögert: das Fehlen einer prospektiven klinischen Validierung, die Notwendigkeit einer behördlichen Genehmigung und das Fehlen einer angemessenen institutionellen Infrastruktur für die systematische Datenerfassung und -verarbeitung. (KONSENS)*

a) Verwendungszweck sowie analytische und klinische Validierung, b) Datenqualität und c) Privatsphäre und Datenschutz gehören zu den sechs zentralen Themenbereichen, die von der WHO für eine sichere und schnelle Nutzung von KI-Systemen im Gesundheitswesen angesprochen werden [9]. Vor allem die Erhebung vielfältiger und für die Zielpopulation repräsentativer Daten ist für die Gewährleistung der „algorithmischen Sicherheit“ unerlässlich [10]. Außerdem sollte das Paradigma des „federated learning“ in Betracht gezogen werden, um den Datenschutz und Fragen der gemeinsamen Nutzung von Daten zu wahren und gleichzeitig die Entwicklung robuster, institutionenübergreifender Modelle zu gewährleisten [11].

*Der demografische Wandel führt durch die alternde Bevölkerung zu einem steigenden Bedarf an Gesundheitsleistungen, den das Gesundheitssystem ohne den Einsatz von KI kaum bewältigen kann. (KONSENS)*

Der Einsatz von KI im Gesundheitswesen birgt vielversprechendes Potenzial, wirft aber auch ethische Bedenken und Herausforderungen auf. Die Integration von KI-Lösungen in die Gesundheitsversorgung älterer Menschen erfordert daher eine sorgfältige Planung und die Berücksichtigung von Aspekten wie externer Validierung, Transparenz und Schutz vor Fehlern. Um den individuellen Bedürfnissen älterer Menschen angemessen Rechnung zu tragen, ist ein personenzentrierter Ansatz unerlässlich [12].

*Die technischen Operationspunkte von Algorithmen zur Detektion pathologischer Befunde in radiologischen Bilddaten balancieren eine häufig priorisiert hohe Sensitivität mit der im Rahmen dessen noch erreichbaren Spezifitäten und klinisch-prädiktiven Werten* [13–15]. *Zwar eignen sich hochsensitive Algorithmen als „second reader“, um die Anzahl übersehener Befunde zu reduzieren* [16–18]. *Jedoch bergen hochsensitive Algorithmen mit resultierend auch falsch-positiven Detektionen in Kombination mit einem möglichen „confirmation bias“ klinischer Anwender die Gefahr, dass Patienten unnötigerweise einer erweiterten Diagnostik und Therapie zugeführt werden* [14, 19, 20]. *(KONSENS)*

Bereits in den 1980er-/1990er-Jahren führten „Computer-aided-design“ (CAD)-Systeme in der Mammographie zu kritischen Diskussionen über unnötige Abklärungen [19, 20] durch (zu) hohe Sensitivitäten und limitierte prädiktive Werte [13, 14, 16–18]. Ähnliche Herausforderungen bringen auch Anwendungen modernerer KI-Algorithmen mit sich [15].

*Der Einsatz von KI-basierten Chatbots zur Beantwortung von Patientenfragen, z.* *B. bei der präoperativen Schulung oder in der postoperativen Nachsorge, beschränkt sich auf wenige Studien. Ebenso ist die KI-gestützte Überwachung postoperativer Fortschritte sowie die frühzeitige Erkennung von Komplikationen nicht Teil der klinischen Routine. (STARKER KONSENS)*

Studienergebnisse, wie z. B. bei der Wissensvermittlung in der Prostatakrebsdiagnostik, zeigen, dass Chatbots von Patienten genutzt und erfolgreich klinisch eingesetzt werden können [21]. Obwohl vielversprechende Studienergebnisse zu beobachten sind, erfordert die Integration in klinische Arbeitsabläufe weitere Forschung, um ethische, technische und regulatorische Herausforderungen zu bewältigen. KI-basierte Chatbots können Ärzte und Patienten unterstützen, ersetzen jedoch nicht die zwischenmenschliche Arzt-Patienten-Beziehung.

*Die KI zur intraoperativen Qualitätssicherung und Verbesserung der Patientensicherheit beschränkt sich derzeit auf wissenschaftliche retrospektive Analysen von operativen Videos, zur Segmentierung von anatomischen Strukturen, Phasenerkennung und Bewertung chirurgischer Fähigkeiten. (KONSENS)*

Obwohl KI-Anwendungen zunehmend in der präoperativen Diagnostik, Therapieplanung, Risikostratifizierung und postoperativen Verlaufskontrolle eingesetzt werden, bleibt ihr Potenzial für intraoperative Qualitätssicherung und Patientensicherheit bislang weitgehend ungenutzt. Aktuelle, überwiegend wissenschaftlich-experimentelle Ansätze zielen primär auf ein automatisiertes Verständnis intraoperativer Abläufe sowie auf die retrospektive Erkennung und Klassifikation räumlicher und zeitlicher Aspekte im endoskopischen Video ab (z. B. Erkennung operativer Phasen, Vorhersage nächster Schritte oder Einordnung von Instrument-Gewebe-Interaktionen) sowie die Bewertung chirurgischer Fähigkeiten [22–27].

*Das Training von KI-System in der Medizin erfordert aktuell große Mengen an Gesundheitsdaten. Gesundheitsdaten werden unter Einsatz einer doppelten Pseudonymisierung durch eine Vertrauensstelle von potenziell identifizierbaren Informationen getrennt. (KONSENS)*

Mit dem am 26.03.2024 in Kraft getretenen Gesundheitsdatennutzungsgesetz (GDNG) besteht der rechtliche Rahmen, medizinische Daten in breiterer Anwendung nutzbar zu machen. Zum aktuellen Zeitpunkt fehlt aber die Infrastruktur, diese Gesundheitsdaten anzufordern, da sich das neue Forschungsdatenzentrum Gesundheit der gesetzlichen Ombudsbehörde, dem Bundesinstitut für Arzneimittel und Medizinprodukte, noch im Aufbau befindet. Anträge werden voraussichtlich ab Anfang 2025 bearbeitet werden können [28–30].

*Zum aktuellen Zeitpunkt bestehen keinerlei gesetzliche Haftungspflicht und Urheberrechte für die Verwendung von medizinischen, KI-generierten Inhalten. Damit Inhalte urheberrechtlich geschützt sind, müssen sie gemäß § 2 Abs. 2 UrhG als „Werk“ gelten, was eine „persönliche geistige Schöpfung“ voraussetzt. Diese Schöpfung muss auf menschlicher kreativer Tätigkeit beruhen, um als „persönlich“ zu gelten. KI-erzeugte Inhalte gelten in der Regel nicht als urheberrechtlich geschützt, da keine „persönliche geistige Schöpfung“ vorliegt. (KONSENS)*

Um Verantwortung übernehmen zu können, muss ein Subjekt im traditionellen Sinne handlungsfähig, also in der Lage sein, Entscheidungen zu treffen. KI erfüllen diese Voraussetzungen nicht, weshalb Verantwortung für deren Verhalten nicht auf die KI selbst übertragen werden kann [31].

*Zu jedem Zeitpunkt unterliegt die Verantwortung der Diagnostik, Diagnosestellung und Therapie, auch bei Verwendung von KI-gestützten Systemen, den behandelnden Ärzten. (KONSENS)*

Für Schnittstellen zwischen Menschen und KI besteht das Risiko einer Verantwortungsdiffusion. Bei der Zuweisung von Verantwortung tritt an die Stelle des Arzt-Patienten-Verhältnisses ein Zusammenspiel von Herstellern, Programmierern, Regulierungsbehörden, medizinischen Anwendenden und Patienten. KI kann selbst nicht für Fehler zur Verantwortung gezogen werden [31]. Akteure sind in der Pflicht, die Verantwortung eindeutig zuzuweisen [32].

*Die aktuellen medizinischen Curricula sind nicht unter Berücksichtigung der hohen Wahrscheinlichkeit, dass in den kommenden Jahrzehnten vermehrt KI-basierte Assistenzsysteme Einzug in die klinische Anwendung halten werden, verfasst worden. (KONSENS)*

Die Inhalte des Medizinstudiums orientieren sich einerseits an den Erfahrungen der bisherigen Lehre, sollen aber gleichzeitig bereits frühzeitig sich abzeichnende, Trends integrieren [33, 34]. Die aktuell für 2027 geplante Novelle der Approbationsordnung stellt eine günstige Gelegenheit dar, es den Studierenden zu ermöglichen, bereits im Rahmen ihrer Ausbildung zu lernen, die Entscheidungsprozesse von KI-Modellen in ihren Grundzügen nachzuvollziehen und dadurch ein Verständnis für Potenzial und Begrenzungen bestimmter Methoden zu entwickeln.

*Aktuell gibt es keine speziell für medizinische Anwendungen trainierte KI-Systeme, die effektiv und zuverlässig für den Wissenserwerb in der medizinischen Ausbildung empfohlen werden können. Die Entwicklung solcher Systeme steht noch am Anfang und bedarf weiterer Forschung und Validierung, bevor sie in der medizinischen Lehre flächendeckend eingesetzt werden können. (KONSENS)*

Obwohl „large language models“ beeindruckende Ergebnisse bei der Beantwortung medizinischer Fragestellungen erbringen, mangelt es aktuell an medizinspezifischem Training, Präzision und Zuverlässigkeit, um sie als vertrauenswürdige Werkzeuge für den Wissenserwerb zu nutzen [35–42]. Sorgfältige Planung, rigorose Testungen, fortlaufende Updates, aktive Wartungen, ethische Überlegungen und Datenschutz sind von entscheidender Bedeutung für die Entwicklung solcher Systeme.

### Utopie (maximale Potenzialausschöpfung)

*Der Datenzugriff, die Datenverarbeitung, der Daten-Output sowie die Speicherung des KI-generierten Inhalts unterliegt nicht der Verantwortung der Ärzte. Die Nutzung und Anwendung dessen für Klinik, Forschung und Lehre jedoch unterliegt der Verantwortung der Ärzte. (KONSENS)*

Hersteller müssen sicherstellen, dass KI-Systeme transparent und verantwortungsvoll genutzt werden können. Patienten sollten Entscheidungsunterstützungssysteme in Grundzügen verstehen können, um einen „informed consent“ zu ermöglichen. Ärzte sollen in der Lage sein, eine Risikobewertung bei der Nutzung eines KI-Systems vorzunehmen. Die moralische und rechtliche Letztverantwortung bei KI-gestützten Anwendungen verbleibt beim behandelnden Fachpersonal. Das Vertrauen in die Arzt-Patienten-Beziehung bleibt somit zentral für den Behandlungserfolg. Das Ziel des KI-Einsatzes in der Medizin soll nicht darin bestehen, medizinisches Fachpersonal zu ersetzen, sondern den Behandlungsprozess zu unterstützen [32].

*Die Ergebnisse der im medizinischen Bereich eingesetzten KI-Tools sollten für Kliniker leicht interpretierbar sein, sie sollten plausible, logische Begründungen für das Ergebnis liefern (auch bei Unsicherheiten) und transparent sein. (KONSENS)*

Die KI-Systeme sollten transparente Entscheidungsprozesse zeigen, um Vertrauen zu schaffen. Vor allem sollten KI-Tools nicht nur Vorhersagen, sondern auch Begründungen liefern, die mit dem medizinischen Wissen übereinstimmen. Plausibilität, gepaart mit Interpretierbarkeit, ist für Kliniker entscheidend, damit sie KI-Ergebnissen vertrauen und sie überprüfen können, insbesondere in kritischen Bereichen wie Diagnostik und Behandlungsplanung. Techniken wie Entscheidungsbäume, „attention maps“ in neuronalen Netzen und „feature importance scores“ können die Interpretierbarkeit verbessern. Die Verankerung von Kompetenzen im Bereich der digitalen Gesundheit in der Aus- und Weiterbildung von Ärzten [43] schafft die Voraussetzungen für eine sachkundige Nutzung und Sensibilisierung für diese Technologien.

*Die KI-Anwendungen können zu einer verbesserten Erkennung von Patienten führen, die von einer chirurgischen Therapie profitieren können. Gleichzeitig könnte durch eine Reduktion von unnötiger invasiver Diagnostik Schaden vom Patienten abgewendet werden. (KONSENS)*

Die Früherkennung von Erkrankungen in chirurgischen Disziplinen kann Behandlungsergebnisse signifikant verbessern [44]. Allerdings ist die diagnostische Genauigkeit vieler Screening-Methoden limitiert. Moderne KI, insbesondere das „Deep Learning“, kann für spezifische Fragestellungen die menschliche Leistungsfähigkeit erreichen [45] und potenziell zu einer verbesserten Erkennung von Patienten führen, die von einer frühzeitigen Therapie profitieren können. Gleichzeitig könnte durch Reduktion von invasiver Diagnostik Schaden vom Patienten abgewendet werden.

*Die KI-Systeme könnten zukünftig personalisierte und präzise Therapieempfehlungen liefern, die die Behandlungsergebnisse erheblich verbessern könnten. (KONSENS)*

Die KI-Systeme haben das Potenzial, durch die Analyse großer Datenmengen präzise Therapieempfehlungen zu generieren, die individualisierte Behandlungspläne ermöglichen. Diese Systeme könnten klinische Daten, genetische Informationen und Patientenpräferenzen verarbeiten, um maßgeschneiderte Therapievorschläge zu bieten, die die Behandlungsergebnisse optimieren. Beispielsweise könnten sie Ärzten dabei helfen, spezifische Medikationen oder Therapien für einzelne Patienten basierend auf deren individuellen Risikoprofilen auszuwählen und so die Sicherheit der Behandlung zu erhöhen [46]. Studien zeigen, dass die Anwendung solcher Systeme in der Krebsbehandlung bereits zu maßgeschneiderten, multidrug-basierten Therapien geführt hat, die Überlebensraten verbessern konnten [47].

*Die KI-gestützten Therapieunterstützungssysteme können klinische Abläufe zeitlich optimieren und einen Beitrag zur optimierten Patientenversorgung leisten, da individuelle Erfahrungsstände des Personals ausgeglichen werden können, einrichtungsübergreifend Erfahrung genutzt werden kann und komplexe Zusammenhänge kompakter dargestellt werden können. (KONSENS)*

Die KI-Methoden sind aufgrund ihrer Rechenleistung in der Lage, Entscheidungen basierend auf einer riesigen Anzahl an Faktoren zu treffen, wodurch sie Empfehlungen optimieren könnten [48]. Durch eine potenziell enorme Menge an medizinischen Daten sind sie in der Lage, personalisierte Vorhersagen zu treffen [49].

*Klinische KI-Software soll barrierefrei mit synthetischen, frei zugänglichen und keine personenbezogenen Informationen enthaltenden Daten trainiert werden, die reale Daten in gleicher Weise abbilden. (KONSENS)*

Die KI ist in der Lage, authentische synthetische Gesundheitsdaten zu erzeugen, die keine personenbezogenen Informationen enthalten [50–53]. Synthetische Daten können ohne datenschutzrechtliche Einschränkungen genutzt werden und bestehende Datensätze erweitern oder ersetzen. Synthetische Daten müssen wiederum initial von KI mithilfe von echten Daten („Big Data“) erzeugt werden [54]. Damit kann u. a. die Genauigkeit KI-basierter Diagnosesysteme verbessert werden und sie sind barrierefrei in der Klinik, Forschung und Lehre etc. einsetzbar, ohne auf Bundesdatenbanken angewiesen zu sein [28, 29].

*Das partizipative ärztliche Aufklärungsgespräch kann durch KI-Systeme nicht ersetzt, sondern nur unterstützt werden. (KONSENS)*

Die KI-Systeme sollten das partizipative ärztliche Aufklärungsgespräch nicht vollständigen ersetzen, obwohl die Systeme das Gespräch durch Informationsvermittlung zu Vorgehensweisen, prozeduralen Abläufen, Risikoeinschätzungen und Formalitäten unterstützen können [55]. Es fehlt die Fähigkeit individuelle Bedürfnisse, Fragen und Emotionen einzuschätzen und deren Konsequenzen in den Entscheidungsprozess einfließen zu lassen [56]. Somit bleibt das Aufklärungsgespräch eine ärztliche Kernkompetenz mit zwischenmenschlicher Interaktion und Empathie, welche nicht durch Technologie ersetzt werden sollte [57].

*Zukünftig sollte KI, unter Verwendung aller in der robotischen Chirurgie verfügbaren Datenmodalitäten (z.* *B. Video und Kinematik), intraoperative Warnsysteme und automatisierte präventive Intervention ermöglichen. (KONSENS)*

Die Vision zielt darauf ab, eine kontinuierliche, datengestützte Überwachung der chirurgischen Umgebung zu ermöglichen, wobei kritische Strukturen hervorgehoben und Bewegungsmuster analysiert werden, um chirurgische Fähigkeiten objektiv zu bewerten und intraoperative Entscheidungen zu verbessern. Insbesondere die robotische Chirurgie bietet hierfür hervorragende Grundvoraussetzungen, da nicht nur visuelle Informationen aufgezeichnet werden, sondern auch kinematische und haptische Daten analysiert werden können [58, 59] und damit intraoperative Warnsysteme und automatisierte präventive Interventionen ermöglicht werden [60, 61]. Zusammengefasst zeigt sich im erzielten Konsensus, dass sowohl aktuelle als auch zukünftige KI-Technologien das Potenzial haben, die robotische Chirurgie sicherer und effizienter zu gestalten.

*Zukünftige KI-Anwendungen sollten intraoperative Echtzeitüberwachung und Bildverarbeitung zur automatischen Fehlererkennung und -vermeidung sowie detaillierte, effiziente Dokumentation ermöglichen. (STARKER KONSENS)*

Der ideale Einsatz von KI für die intraoperative Qualitätssicherung und Verbesserung der Patientensicherheit wird in der automatisierten Erkennung von operativen Risiken und zeitgerechten Warnung bzw. Intervention zur rechtzeitigen Vermeidung von operativen Komplikationen gesehen. Solche Systeme könnten beispielsweise kritische anatomische Strukturen in Echtzeit markieren oder bei fehlerhaften Instrumentenbewegungen Warnungen ausgeben [22, 62]. Darüber hinaus würde eine KI-gestützte Dokumentation den operativen Ablauf automatisch aufzeichnen und analysieren, wodurch nicht nur der administrative Aufwand reduziert, sondern auch wertvolle Daten für zukünftige Operationen generiert werden könnten. Der Weg zu diesem Idealzustand erfordert jedoch erhebliche Fortschritte im Datenmanagement, vorhandener Technologie und regulatorischen Rahmenbedingungen.

*Die verschiedenen Schweregrade unerwünschter Arzneimittelwirkungen (UAW) werden in klinischen Studien hinsichtlich ihrer Häufigkeit berichtet, jedoch oft ohne individualisierbare oder für eine KI-prädiktive Analyse verwertbare statistische Zusammenhänge. Perspektivisch soll die KI-gestützte Vorhersage von UAW durch eine Verbesserung der Datenverfügbarkeit für die wissenschaftliche Öffentlichkeit ermöglicht werden, um eine Optimierung der Therapie zu erreichen. (KONSENS)*

Primäre Endpunkte der Zulassungsstudien konzentrieren sich auf onkologische Endpunkte wie Gesamtüberleben, progressionsfreies Überleben und die Zeit bis zur Folgetherapie. Die Abhängigkeit dieser Endpunkte von klinischen Parametern wird gewöhnlich im Kontext von Subgruppenanalysen erörtert. Toxizitäten werden hinsichtlich der Häufigkeit und der unterschiedlichen Schweregrade berichtet, jedoch in der Regel ohne die Durchführung von Subgruppenanalysen oder die Etablierung statistisch signifikanter Zusammenhänge zu patientenbezogenen Parametern. Für die zukünftige prädiktive Modellierung von UAW unter Verwendung maschineller Lernverfahren sollten die benötigten Rohdaten sowie relevante Informationen für potenzielles „Feature Engineering“ – analog zu den Analysen zur onkologischen Wirksamkeit – im Rahmen einer Subgruppenanalyse bereitgestellt werden.

*Zur zielgerichteten Wissensvermittlung für Patienten könnten sorgfältig validierte KI-Systeme entwickelt und unter der Aufsicht eines Arztes eingesetzt werden. Langfristig könnten diese eine maßgeschneiderte Schulung für Patienten anbieten, die auf die individuellen Bedürfnisse und Gesundheitszustände der Patienten zugeschnitten sind. (KONSENS)*

Die KI kann dabei helfen, die Gesundheitskompetenz der Patienten zu verbessern und individuell zugeschnittene Informationen bereitzustellen. Eine entscheidende Rolle spielt die Supervision durch Ärzte, um sicherzustellen, dass die Technologie korrekt angewendet wird und keine Risiken für die Patienten entstehen. Langfristig könnte KI eine zentrale Rolle in der Unterstützung der personalisierten Patientenbetreuung spielen, wenn Ethik und Datenschutz bei ihrer Implementierung berücksichtigt werden [63].

*Die KI-Systeme können zukünftig die Wissensbeschaffung wesentlich erleichtern und optimieren, wodurch sich Auszubildende vermehrt auf den Wissenserwerb konzentrieren können. (KONSENS)*

Das „Deep-Learning-Konzept“ ermöglicht KI-Modellen, sehr große Datenmengen effizient zu verarbeiten, zu analysieren und aufzubereiten [64, 65]. KI-gestützte Lernsysteme könnten den Wissenszugang revolutionieren, in dem sie das Navigieren durch umfangreiche medizinische Literatur erleichtern. Sie können adaptive Anweisungen sowie multimodale Inhalte bereitstellen und sich an individuelle Lernbedürfnisse anpassen. Durch kognitive Modellierung und effektives Feedback optimieren sie den Lernprozess, während interaktive Szenarien praxisnahe Erfahrungen ermöglichen. Diese Ansätze verbessern nicht nur die Wissensaneignung, sondern auch Selbstreflexion und Lernstrategien, wodurch der Lernerfolg langfristig gesteigert werden könnte [66].

*Durch den Einsatz von KI-basierten Lehrmethoden soll die medizinische Lehre skalierbar weiter personalisiert und letztendlich der individuelle Lernerfolg gesteigert werden. (KONSENS)*

Eine möglichst personalisierte Lehre erhöht gerade im Medizinstudium den Lernerfolg signifikant [67–69]. Gleichzeitig ist das Studium der Humanmedizin einer der teuersten Studiengänge in Deutschland [70] und die medizinischen Fakultäten operieren bereits am oberen Rand ihrer personellen und finanziellen Kapazitäten. Der Einsatz von KI-basierten Tools wie z. B. Sprachmodellen stellt eine sehr valide Möglichkeit dar, um die Qualität der Lehre und damit die Wissensretention und den Fähigkeitserwerb zu erhöhen, ohne dabei die Kosten des Studiums per capita wesentlich zu steigern [71–73].

### Digitale Transformation (vom Status quo zur maximalen Potenzialausschöpfung)

*Es ist eine interdisziplinäre Bündelung aller Ressourcen erforderlich, die es ermöglicht, durch Förderung von Kooperationsnetzwerken zwischen Medizinern verschiedener Fachdisziplinen, Informatikern und anderen Experten auf dem Gebiet der digitalen Medizin, valide KI-Modelle zu entwickeln. (STARKER KONSENS)*

Die Entwicklung von KI-Anwendungen für die Medizin erfordert die Kooperation zwischen Spezialisten aus sehr unterschiedlichen Fachrichtungen. Dies erfordert interdisziplinäres Arbeiten, das direktes Feedback ermöglicht, aber auch Fort- und Weiterbildungsarbeit, um entsprechendes fachfremdes Wissen grundlegend zu beherrschen und ein Verständnis für die speziellen Anforderungen zu entwickeln [74].

Hierbei sollte die technische Entwicklung mit der praktischen in Einklang gebracht werden. Dieses sollte bereits in der Planungsphase gewährleistet sein, um praktisch relevante Modelle für den klinischen Nutzen zu entwickeln. Auf diese Weise wird es möglich, dass sich KI-Tools mit der klinischen Praxis weiterentwickeln und die diagnostische Genauigkeit und Effizienz verbessern.

*Durch eine idealerweise einrichtungsübergreifende Standardisierung der klinischen Prozesse und Dokumentationen könnte die Entwicklung KI-basierter Unterstützungssysteme inklusive Sprachmodellen begünstigt werden, da Engpässe bezüglich der Datenverfügbarkeit, des Datenaustausches und der Datenstandardisierung besser adressiert wären. (KONSENS)*

Daten werden durch Standardisierung interoperabel und erst dadurch analysierbar [75, 76]. Inkonsistente Daten oder Daten mit fehlenden Werten erschweren das Training von KI-Methoden. Ebenso wird in beiden Publikationen darauf hingewiesen, dass eine stärkere Zusammenarbeit zwischen Medizinern und Informatikern dazu beitragen kann, eine Repräsentation der Daten zu finden, welche eine effiziente Verarbeitung mittels KI-Methoden und einen Datenaustausch ermöglicht.

*Es sollten spezielle Sprachmodelle trainiert werden, die objektive Gesundheitsdaten trotz individueller Textformulierungen sicher verarbeiten können. (STARKER KONSENS)*

Die Komplexität und Menge von Patientendaten, die abteilungsspezifisch und damit heterogen dokumentiert werden, erfordert eine sichere und vollständige Erkennung der relevanten Informationen [77]. Das aktuell hohe Risiko für Halluzinationen einerseits und Mängeln bei der Befunderkennung durch KI andererseits begünstigt Fehlinterpretationen [78], die mit steigender Qualität der Modelle reduziert werden.

*Der Überführung diagnostischer KI-Algorithmen zur Analyse radiologischer Bildgebung in die klinische Routine sollen externe Validierungsstudien vorausgehen* [79–82]. *Diese Validierungsstudien sollten spezifisch den klinischen Anwendungsfall an repräsentativen Patientenkohorten *[80, 83, 84] *simulieren, die Algorithmus-Performance an einem robusten Referenzstandard (z.* *B. Konsensus-Reading mehrerer im jeweiligen Anwendungsfeld erfahrener radiologischer Fachärzte* [80, 85–87]*)* *messen und auch auf mögliche „Confounder“* [88–92] *in den zugrunde liegenden Trainingsdaten testen, um systematische Fehler zu vermeiden und die Robustheit der Algorithmen zu gewährleisten* [92]. *(KONSENS)*

Diese Validierungsstudien müssen robuste Referenzstandards [79–82, 85–87] nutzen, auf „Confounder“ [88–92] prüfen und an realen Patientenkohorten [80, 83, 84] durchgeführt werden, um systematische Fehler zu vermeiden [92].

*Bei der Implementierung und Nutzung diagnostischer Algorithmen zur Analyse radiologischer Bildgebung sollten auch potenziell negativen Auswirkungen sorgfältig berücksichtigt werden und Maßnahmen (ggf. auch instituts-/klinikspezifisch) ergriffen werden, um diese Risiken zu minimieren. (KONSENS)*

Um klinische Risiken durch den Einsatz von KI zu minimieren, sollten prospektive Studien durchgeführt werden, die auch klinische Endpunkte einbeziehen. Diese Studien bieten die Möglichkeit, die tatsächlichen Auswirkungen auf Patienten und mögliche negative Effekte wie Fehldiagnosen besser zu verstehen [19, 93].

*Die Ausgabe eines KI-Algorithmus sollte dem Arzt verständlich erklärt werden sowie dessen Unsicherheit mitgeteilt werden, anstelle einer simplifizierenden Ja-oder-Nein-Antwort, damit die Ausgabe ärztlich auditiert, und an den Patienten kommuniziert werden kann. (KONSENS)*

Ein KI-Algorithmus sollte dem Anwender die individuelle Ausgabe erklären können, um so Transparenz und Verständnis zu schaffen („explainable AI“). Dies kann erreicht werden durch die Verwendung intrinsisch erklärbarer Modelle wie linearer Modelle oder Entscheidungsbäume. Alternativ können auch Surrogatmodelle wie LIME („local interpretable model-agnostic explanations“) oder Methoden aus der kooperativen Spieltheorie wie SHAP („shapley additive explanations“) eingesetzt werden, um die Entscheidungsfindung des Modells nachvollziehen zu können.

Ein KI-Algorithmus sollte zudem eine Einschätzung seiner Unsicherheit bezüglich der Ausgabe liefern. Dies könnte durch eine probabilistische Kalibrierung an die Zielpopulation erfolgen, beispielsweise in Form von Konfidenzintervallen für Regressionsaufgaben oder Wahrscheinlichkeiten für Klassifizierungsaufgaben. Ein geeignetes Framework hierfür bietet „Conformal Prediction“ [94, 95].

*Modelle des maschinellen Lernens (ML) sollen implementiert werden, um UAW frühzeitig zu identifizieren oder vorherzusagen. Diese Modelle sind kontinuierlich zu aktualisieren und zu validieren. (STARKER KONSENS)*

Die Implementierung dieser Modelle in der Medizin wird zunehmend als transformative Methode anerkannt. ML kann z. B. erfolgreich den Behandlungserfolg bei Psoriasispatienten vorhersagen, wobei eine Genauigkeit von > 80 % bei der Vorhersage des Abbruchs von Medikamenten aufgrund unerwünschter Ereignisse oder Verlust der Wirksamkeit erreicht wurde [96]. Die kontinuierliche Aktualisierung und Validierung dieser Modelle ist entscheidend, um ihre Relevanz und Genauigkeit in einem sich schnell wandelnden Gesundheitsumfeld aufrechtzuerhalten. Ross et al. betonten die Bedeutung von Echtzeitprognoseanalysen aus elektronischen Patientenakten, was eine fortlaufende Verfeinerung der Modelle erfordert, um sich an neue Daten und aufkommende Muster anzupassen [97].

*Um Patienten zielgerichteter mit Informationen zu versorgen, soll die Entwicklung und Implementierung von KI-gestützten Chatbots vorangetrieben werden. Dabei soll die Integration in bestehende Versorgungsstrukturen gewährleistet sowie die Datensicherheit und der Datenschutz sichergestellt werden. (KONSENS)*

Die Entwicklung von KI-gestützten Chatbots bietet großes Potenzial, um Patienten schnell, effizient und personalisiert mit Gesundheitsinformationen zu versorgen. Eine erfolgreiche Implementierung setzt voraus, dass die Chatbots für die Patienten benutzerfreundlich sind und sich nahtlos in bestehende Versorgungsstrukturen integrieren lassen, um die Arbeitsabläufe von Ärzten und Pflegepersonal zu unterstützen [98]. Entscheidend ist auch, dass die Datensicherheit und der Schutz sensibler Gesundheitsdaten durch sorgfältige Regulierung und Verschlüsselungstechnologien gewährleistet werden.

*Um KI effizient und zuverlässig zur Verbesserung der Patientensicherheit und chirurgischen Qualität einzusetzen, müssen krankenhauspolitische und rechtliche Voraussetzungen geschaffen werden, welche die weitreichende Nutzung von multizentrischen klinischen sowie Video- und Bilddatensätzen für das Training und die Validierung von KI-Modellen unterstützen. (KONSENS)*

Neben einer umfassenden technischen und regulatorischen Infrastruktur bedarf die klinische Implementierung von KI in intraoperative chirurgische Prozesse v. a. umfassende klinische Studien zur Validierung der Sicherheit, Zuverlässigkeit und Relevanz der Modelle. KI-Methoden sollten im Kontext realer Entscheidungsszenarien und klinischer Outcomes evaluiert werden, um die Praxistauglichkeit sicherzustellen.

*Der klinische Einsatz von KI zur Qualitätssicherung und Verbesserung der Patientensicherheit in der Chirurgie erfordert die zunehmende Einbeziehung aller Interessensgruppen. Dies schließt Vertreter von Datenschutz, Informationstechnik und Industrie sowie aktuell unterrepräsentierte Gruppen wie Pflegekräften, Verwaltung, Medizinstudenten, Datenschutzvertreten und Patienten mit ein. (KONSENS)*

Neben chirurgischen Experten, die die klinische Relevanz von KI bewerten und deren zielgerichtete Entwicklung mitbeeinflussen sowie Computerwissenschaftlern, die adäquate technische Lösungen entwickeln, bedarf es diverser anderer Interessensgruppen, um eine klinisch fundierte KI zu entwickeln. Neben administrativen und juristischen Fachkräften, um datenschutzrechtliche und regulatorische Anforderungen zu adressieren [99, 100], bedarf es v. a. auch der Einbindung von Patienten und Pflegekräften, die die Nutzung von KI im klinischen Rahmen bewerten. Ethische, moralische und kulturelle Aspekte sollten berücksichtigt werden, um den Weg für die klinische Implementierung zu ebnen. Mit der zunehmenden Komplexität und der fortschreitenden Integration von KI in die klinische Praxis wächst die Liste der Interessensgruppen stetig. Dies sichert nicht nur die Entwicklung robuster KI-Modelle, sondern trägt auch zur Akzeptanz und Sicherheit von KI-gestützten Anwendungen in der Chirurgie bei.

*Die Unterstützung durch Politik und öffentliche Krankenhäuser ist entscheidend für die Entwicklung und Implementierung von KI-Modellen im Gesundheitswesen. (KONSENS)*

Politische Unterstützung ist ausschlaggebend, um die klinische Anwendung von KI voranzutreiben und deren Potenzial zur Verbesserung der Gesundheitsversorgung auszuschöpfen. Die Schaffung regulatorischer Rahmenbedingungen, Bereitstellung notwendiger Ressourcen, Innovationsförderung, Stärkung der Zusammenarbeit von „Stakeholdern“, Etablierung von Datenschutzrichtlinien und ethischen Standards sollten gefördert werden [101, 102]. Öffentliche Krankenhäuser bieten eine ideale Plattform für die Entwicklung und Implementierung von KI-Technologien, da sie über große und diverse Patientendaten verfügen und in der Praxis validierte Modelle benötigen. Ein ganzheitlicher Ansatz ist notwendig, um langfristige Verbesserungen zu erzielen [103].

## Diskussion

Der erarbeitete Delphi-Konsensusprozess für KI-Anwendungen in der Chirurgie zeigt eindrücklich, welche großen Chancen KI für eine verbesserte Patientenversorgung darstellt. Die Anwendungen umfassen nahezu alle Bereiche der chirurgischen Behandlungsabläufe, von der Prävention über die Diagnostik, Aufklärung bis hin zur Therapie und Nachsorge der Patienten. Um dieses enorme Potenzial zu verwirklichen, wurden im Zuge des systematischen Delphi-Konsensusprozesses bestehende Limitationen identifiziert, die insbesondere auf die Verbesserung einheitlicher Digitalisierungsstrukturen sowie die rechtlichen Rahmenbedingungen fokussieren. Die interdisziplinäre Expertengruppe hat hieraus konkrete und praxisnahe Vorschläge für eine erfolgreiche Transformation erstellt, die alle verantwortlichen Akteure in der Medizin, Politik und Wirtschaft adressiert. Limitation dieses Delphi-Konsensus ist die relativ geringe Anzahl von 16 Akteuren, die jedoch durch die breite interdisziplinäre Zusammensetzung sowie die intensiven und konstruktiven Diskussionen in diesem kleinen Rahmen ausbalanciert wurde. Wir appellieren daran, die entwickelten Lösungsansätze zukünftig ebenso interdisziplinär zu erarbeiten und zu beraten, um im internationalen Vergleich und Wettbewerb das deutsche Gesundheitswesen auch weiterhin zukunftsfähig in die digitale Transformation zu führen.

## Fazit für die Praxis

- Künstliche Intelligenz (KI) hat bedeutendes Potenzial, die Patientenversorgung in allen Bereichen chirurgischer und angeschlossener Fachrichtungen zu verbessern.
- KI-basierte Gesundheitslösungen sollten von Grund auf multidisziplinär konzipiert und entwickelt werden. Dazu gehören u. a. medizinische Leistungserbringer, Medizintechnik und Pharmaindustrie, digitale Medienpräsenz, Patientenvertreter und Experten aus den Bereichen Ethik und Recht.
- Für die nationale klinische Anwendung von KI-basierten Assistenzsystemen stellen die rechtlichen Verwaltungsstrukturen des Föderalismus eine komplexe politisch-strukturelle Herausforderung dar. Dies betrifft insbesondere die Umsetzung von Datenschutzvorgaben, Informationssicherheit sowie bestehende heterogene Digitalisierungsstandards.
- Es besteht die medizinische Notwendigkeit, dass die Verantwortung für Diagnostik, Diagnosestellung und Therapie, auch bei der Verwendung KI-gestützter Systeme, zu jeder Zeit den behandelnden Ärzten unterliegt.

## Data Availability

Detaillierte Daten zur Konsensstärke der einzelnen Statements können auf Anfrage eingesehen werden.
